# Effect of meal composition on postprandial lipid concentrations and lipoprotein particle numbers: A randomized cross-over study

**DOI:** 10.1371/journal.pone.0172732

**Published:** 2017-02-21

**Authors:** Meena Shah, Manall Jaffery, Beverley Adams-Huet, Brian Franklin, Jonathan Oliver, Joel Mitchell

**Affiliations:** 1 Department of Kinesiology, Texas Christian University, Fort Worth, Texas, United States of America; 2 Department of Clinical Sciences, University of Texas Southwestern Medical Center at Dallas, Dallas, Texas, United States of America; Universita degli Studi di Milano, ITALY

## Abstract

**Background:**

It is unclear how high-protein (HP) and high-monounsaturated fat (HMF) meals affect postprandial blood lipids and lipoprotein particle numbers (LPN).

**Purpose:**

To compare a HP versus a HMF meal on postprandial lipid and LPN responses.

**Methods:**

Twenty-four participants (age: 36.3±15.0 years; body mass index: 23.6±2.0 kg/m^2^; 45.8% female) were fed a HP (31.9% energy from protein) and a HMF (35.2% fat and 20.7% monounsaturated fat) meal in a randomized cross-over trial design. Energy and carbohydrate content were the same across meals. Blood samples were drawn in the fasting state and 3 hour postprandial state, and assessed for lipids and LPN.

**Results:**

Repeated measures analysis showed a significant (p<0.05) treatment by time interaction effect for triglycerides (TG), the primary variable, total high-density lipoprotein particles (T-HDLP) and T-HDLP minus large-buoyant high-density lipoprotein 2b (T-HDLP—LB-HDL2b). HP versus HMF condition led to significantly lower TG at 120 (geometric mean: 90.1 (95% confidence interval (CI): 76.4–106.3) vs. 146.5 (124.2–172.9) mg/dL) and 180 (101.4 (83.1–123.8) vs. 148.7 (121.9–181.4) mg/dL) min and higher T-HDLP at 120 (mean difference: 297.3 (95% CI: 48.6–545.9) nmol/L) and 180 (291.6 (15.8–567.5) nmol/L) min. The difference in T-HDLP by condition was due to the significantly higher small-dense HDLP (T-HDLP—LB-HDL2b) during HP versus HMF condition at 120 (mean difference: 452.6 (95% CI: 177.4–727.9) nmol/L) and 180 (496.8 (263.1–730.6) nmol/L) min. Area under the curve analysis showed that HP versus HMF condition led to significantly lower TG, non-HDLP, and very-low-density lipoprotein particles (VLDLP) responses but significantly less favorable responses in LB-HDL2b particles, T-HDLP—LB-HDL2b, and LB-HDL2b/T-HDLP ratio.

**Conclusion:**

The HP meal led to lower TG, non-HDLP, and VLDLP but less favorable LB-HDL2b, small-dense HDLP, and LB-HDL2b/T-HDLP ratio responses versus a HMF meal. Further studies are needed to confirm these findings over multiple meals.

## Introduction

Replacing high-carbohydrate diets with either high-protein (HP) [1–4] or high-monounsaturated fat (HMF) [2, 5, 6] diets has been found to improve certain blood lipid concentrations. What is not well understood, however, is how a HP intake affects blood lipids compared to a HMF intake, while controlling for carbohydrate intake.

Only two studies have compared the effect of a HMF with a HP diet on blood lipids. Appel et al. [2] found that compared to a HMF diet, a HP diet of similar carbohydrate content, significantly lowered fasting blood total cholesterol (TC) and triglycerides (TG) but worsened high-density lipoprotein cholesterol (HDLC) and did not lower low-density lipoprotein cholesterol (LDLC) or non-HDLC. Luscombe-Marsh et al. [7], on the other hand, found that carbohydrate restricted HP or HMF diets given during 12 wk of energy restriction and 4 wk of energy balance were not different in improving TC, LDLC, TG, and HDLC concentrations.

Both Appel et al. [2] and Luscombe-Marsh et al. [7] measured fasting but not postprandial lipid responses to the two diets. Postprandial lipid concentrations are important to examine since postprandial lipid responses are predictors of coronary artery disease risk [8], and most individuals living in Western societies are in a postprandial state for most of the day [9]. In addition, according to a recent review by Jacome-Sosa et al. [10], not much is known about how acute daily activities such as eating affect the metabolic responses after a meal, and future studies should focus on the postprandial metabolism of nutrients. Another limitation is that neither Appel et al. [2] nor Luscombe-Marsh et al. [7], reported any data on lipoprotein particle density and numbers. Remnant lipoproteins particles and small-dense LDL particles are associated with a greater risk whereas large-buoyant HDL particles are associated with a lower risk for cardiovascular disease [11–14]. In addition to these limitations, the carbohydrate content of the two diets in the study by Luscombe-Marsh et al. [7] was low (35–36% energy from carbohydrate) and probably unsustainable over the long term. The protein rich diet by Appel et al. [2] consisted of only 25% of total energy intake from protein. This is much lower than the upper limit of 35% energy from protein established by the Food and Nutrition Board, Institute of Medicine [15, 16].

The objective of this study was to compare the effect of a HP versus HMF meal on postprandial lipid concentrations and lipoprotein particle numbers while addressing the limitations identified above. The primary variable was TG concentration and the secondary variables were lipoprotein cholesterol concentrations and lipoprotein particle numbers.

## Materials and methods

### Participants

Twenty four participants, ages 18 through 65 years, completed the study. Individuals were excluded from the study if they met any of the following criteria: using agents that lower body weight or blood glucose, dieting to lose weight, being a vegan, lactose intolerance, liver, kidney, gastrointestinal, adrenal, or untreated thyroid disease, diabetes, previous bowel surgery, documented malabsorption, heavy drinking (≥ 14 drinks/wk in men and ≥ 7 drinks/wk in women), smoking, pregnancy, lactation, severe depression, eating disorders, documented malabsorption, or bowel surgery that affects absorption. Because of difficulty in recruiting subjects, the current study deviated from the original protocol of excluding individuals who were on lipid lowering medications. Two subjects with lipid lowering medication were included in the study. They were on the same medication and dose during each meal condition. Including these participants may make the study sample more applicable to the general population. A sensitivity analysis excluding them from the analysis did not change the study outcomes.

The study was approved by the Institutional Review Board of Texas Christian University (TCU). Informed consent was read and signed by all participants before taking part in the study. The study was conducted in accordance to the principles expressed in the Declaration of Helsinki and by the Office for Human Research Protection, U.S. Department of Health and Human Services. Data collection took place in the Metabolic and Exercise Physiology Laboratories at TCU from October 2015 to April 2016. This trial was registered at clinicaltrials.gov (NCT02529709). The CONSORT checklist (S1 File), the study protocol (S2 File), and the data file (S3 File) are provided as supporting information.

### Experimental design

The effect of a HP versus a HMF meal on postprandial blood lipid concentrations and lipoprotein particle numbers were studied using a randomized cross-over trial design. Each participant came to the research center on two days after an overnight fast. On day 1, the participant ate either a HMF or HP test meal in a random order. On day 2, after a washout period of 3 or more days, the participant consumed the alternate meal. Blood samples were drawn in a 12-hour fasted state immediately prior to meal consumption and in the postprandial state over 3 hours.

One of the authors (BAH) used blocked randomization to determine the meal condition sequence, and another author (MJ) enrolled and assigned participants to the meal conditions. The participants were not informed which meal condition they received during each study day.

### Test meals

The HMF meal consisted of 35.2% energy from total fat, 20.7% from monounsaturated fat, 12.6% from protein, 8.7% from saturated fat, and 52.3% from carbohydrates. The HP meal consisted of 31.9% energy from protein, 15.5% from total fat, 4.3% from monounsaturated fat, 9.9% from saturated fat, and 52.6% from carbohydrates. Both the meals were served as beverages. The HMF meal was prepared with plain low-fat yogurt, avocado, and sugar and the HP meal with non-fat Greek yogurt, plain whole milk yogurt, and sugar. The two meals had the same energy content, volume, and added sugar quantity. The meals for male participants contained 840 kcal and those for female participants contained 700 kcal. These values are about 35% of total daily energy needs for men and women, respectively.

### Study protocol

Each participant was asked to come to the research unit at the same time on both days after a 12-hour overnight fast. Female participants with a menstrual cycle were scheduled to consume both meals during the follicular stage of their cycle.

The participants were instructed to consume the same diet on the day before the two study days. They were also instructed not to engage in any exercise on the day before the study days. Twenty-four hour food and physical activity recalls were administered to monitor diet and physical activity on the day before the study days and reported in the results. Body weight was measured before the participant received the test meals.

Each participant was instructed to finish the test meals within 20 min and to take the same amount of time consuming both the HP and HMF meals. Water intake during the postprandial period was controlled. The participants were not allowed to drink or eat anything else during the 3-hour postprandial period.

After inserting a venous catheter in the antecubital vein, blood samples were collected in the fasting state before meal consumption and at 30, 60, 120, and 180 min from when meal consumption began. This was done with the participant lying down. Blood samples were centrifuged and plasma was stored at -80°C until analysis. The participants remained sedentary throughout the postprandial blood sampling period.

### Measures

#### Demographics and anthropometry

Information on demographics was obtained via a questionnaire. BMI was computed from measured weight and height (kg/m^2^). Percent body fat was measured by dual-energy x-ray absorptiometry procedure. Waist circumference was measured at the level of the umbilicus.

#### Exercise and diet recall

A 24-hour diet recall and 24-hour physical activity recall (adapted from a 7-day food recall) were administered to evaluate the type and amount of food consumed and how much exercise the participants had performed on the day before the study days. Both the 24-hour recall and physical activity recall are valid tools for assessing diet and physical activity, respectively [17, 18]. Energy intake was assessed from the 24-hour diet recall using the Food Processor software program (SQL edition, Salem, OR).

#### Lipid and lipoprotein particle numbers determination

Lipids concentrations and lipoprotein particle numbers were determined by a Clinical Laboratory Improvement Amendments (CLIA) certified and New York State certified laboratory, SpectraCell Laboratories (Houston, Texas). Lipids were measured by SpectraCell Laboratories using commercially available enzymatic kits (Beckman Coulter, Indianapolis, IN). Lipid concentrations were measured at 0 (fasting), 30, 60, 120, and 180 min. Lipid measurements included TC, LDL-C, HDLC, and TG concentrations. Non-HDLC was computed by subtracting HDLC from TC and TC:HDLC ratio was calculated by dividing TC by HDLC.

Lipoprotein particle numbers were measured at 0, 120, and 180 min. Lipoprotein particle numbers were analyzed using the lipoprotein subgroup particle number determination technique by SpectraCell Laboratories. The procedure is described in a patent (Patent No.: US 7,856,323 B2) and elsewhere [19] and uses a continuous gradient produced by analytical ultracentrifugation. This method is carefully calibrated and the values agree with other certified methods.

With this technique, particles including very low-density lipoprotein particles (VLDLP), remnant lipoprotein particles (RLP), total low-density lipoprotein particles (T-LDLP), non-high-density lipoprotein particles (non-HDLP), small-dense low-density lipoprotein III (SD-LDL III) particles, small-dense low-density lipoprotein IV (SD-LDL IV) particles, total high-density lipoprotein particles (T-HDLP), and large-buoyant HDL 2b (LB-HDL2b) particles were determined. The presence of small-dense HDL particles was determined by subtracting LB-HDL2b from T-HDLP (T-HDLP—LB-HDL2b). Also computed was the ratio between LB-HDL2b and T-HDLP (LB-HDL2b/T-HDLP). The coefficient of variation for the lipoprotein particle analysis was reported as 5%.

### Sample size and statistical analysis

A sample size of 24 participants was planned a-priori but without a formal sample size calculation. A post-hoc power calculation was not performed.

Demographic and anthropometric characteristics and use of cholesterol lowering medications of the participants are presented by gender and for the entire sample. The data are presented as number of participants and percentages for categorical variables and means and standard deviations for continuous variables.

Baseline values of the participants, i.e., the fasting lipids, body weight, and energy intake and exercise duration over 24-hours before meal consumption were presented by the study period. The differences in baseline values by study period were assessed by paired t-test for normally distributed variables (TC, LDLC, HDLC, non-HDLC, TC:HDLC ratio, and body weight) and Wilcoxon test for variables that were not normally distributed (energy intake and exercise duration over 24 hours prior to meal consumption, and TG).

The effects of meal condition, time, and the interaction between these factors on lipid concentrations and lipoprotein particle numbers were analyzed by mixed-effects model repeated measures analysis. This was the primary analysis of outcome measures. Post-hoc comparisons were performed by computing least square mean contrasts. Triglycerides and VLDLP were not normally distributed and were log transformed before being analyzed. However, TG and VLDLP were back transformed to original units and presented as geometric means and 95% confidence intervals (CI) for ease of interpretation. The remaining variables were presented as arithmetic means and 95% CI. The meal sequence and the interaction between meal and sequence effects were also assessed in the mixed-effects models.

Mixed-effects model was also used to evaluate the effect of meal condition, time, and the interaction between these factors on TG after excluding the two participants on lipid lowering medications.

Area under the curve (AUC) was determined for all the lipid concentrations and lipoprotein particle numbers as the total area under the curve using the trapezoidal rule (AUC = ((s_1_+s_2_/2)*t_1_) + ((s_2_+s_3_/2)*t_2_) + …; “s” = blood concentration of a lipid or lipoprotein particle number and “t” = the elapsed time in min.) Repeated measures analysis was used to compare the AUC by meal condition. This was the secondary analysis of outcome measures. TG and VLDLP AUC were log transformed before being analyzed. AUC data were presented as medians, 25^th^ and 75^th^ percentiles and 95^th^ percentiles in the figures. They were also shown as geometric means and 95% CI for TG and VLDLP and as mean differences and 95% CI for the remaining variables.

P value <0.05 was considered to be significant. All analyses were conducted on 24 participants and using SAS statistical package version 9.4 (SAS Institute, Cary NC).

## Results

### Flow chart

**Fig 1:**
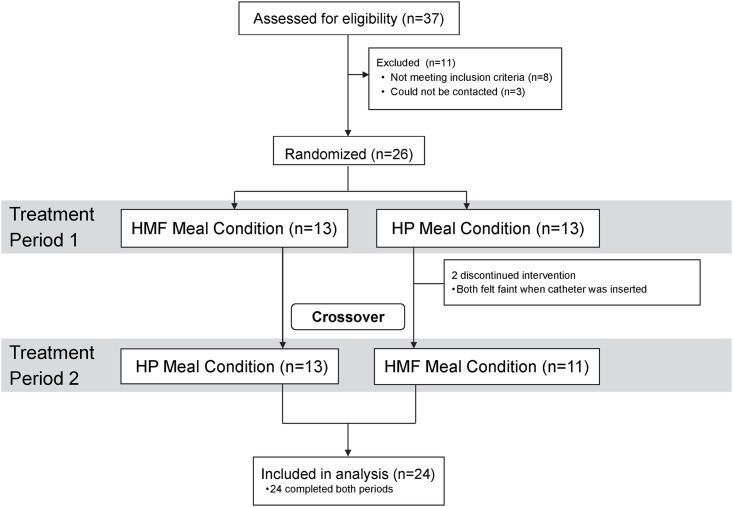
Consort diagram. The number of participants evaluated for eligibility, excluded, randomized, completed the study, and included in the statistical analysis. Abbreviations: HP, high-protein; HMF, high-monounsaturated fat.

The study consort diagram is shown in Fig 1. Thirty-seven individuals were screened for eligibility. Eleven individuals were not included in the study (one was pregnant, one had eating disorders, 4 were trying to lose weight, one was taking testosterone, one was older than 65 years, and 3 had difficulty scheduling their study days). Twenty-six participants were found eligible for the study. Thirteen participants were randomized to the HP condition first. Two of these participants dropped out because they felt dizzy when the catheter was inserted. Eleven participants completed the HP condition and crossed over and completed the HMF condition. Thirteen participants were randomized to the HMF meal condition first. All 13 participants completed the HMF meal condition and crossed over and completed the HP meal condition. A total of 24 participants completed both the meal conditions. Data on twenty-four participants were included in the statistical analysis.

### Participant characteristics

Participant characteristics are presented in Table 1 by gender and for the total sample. The data are shown by gender because percent body fat and waist circumference vary by sex. Mean (SD) age of the total sample was 36.3 ± 15.0 years. Nearly 46% of the sample was female, 42% Hispanic or Latino, and 21% minorities. Mean body mass index, percent body fat, and waist circumference were within the normal levels. Two subjects were on lipid lowering medications throughout the study, and the sequence of randomization to the meal conditions was HP/HMF for one subject and HMF/HP for the other subject.

**Table 1:** Socio-Demographic and anthropometric characteristics of the subjects.

Variables	Males (n = 13)	Females (n = 11)	All Subjects (n = 24)
**Age (year)**	32.9 ± 13.4	40.2 ± 16.3	36.3 ± 15.0
**Ethnicity**			
• Hispanic or Latino	5 (38.5%)	5 (45.5%)	10 (41.7%)
• Non- Hispanic	8 (61.5%)	6 (54.5%)	14 (58.3%)
**Race**			
• Black	0 (0%)	1 (9.1%)	1 (4.2%)
• Asian	2 (15.4%)	1 (9.1%)	3 (12.5%)
• White	10 (76.9)	9 (81.8%)	19 (79.2%)
• Other	1 (7.7%)	0 (0%)	1 (4.2%)
**Body mass index (kg/m**^**2**^**)**	24.5 ± 1.9	22.5 ± 1.6	23.6 ± 2.0
**Percent body fat (%)**	21.3 ± 6.1	31.9 ± 7.5	26.1 ± 8.6
**Waist circumference (cm)**	86.6 ± 5.0	76.2 ± 6.1	81.8 ± 7.5
**Use of lipid medications**	1 (7.7%)	1 (9.1%)	2 (8.3%)

The data are presented as number of subjects and percentages for categorical variables and means and standard deviations for continuous variables.

### Baseline variables

Baseline variables are shown by study period in Table 2. The data are presented as means and standard deviations for normally distributed variables (TC, LDLC, HDLC, non-HDLC, TC:HDLC ratio, and body weight) and medians and 25^th^ and 75^th^ percentiles for variables that were not normally distributed (energy intake and exercise duration over 24 hours prior to meal consumption, and TG). TG are also presented as log values and geometric means and 95% CI. Fasting lipids, body weight, and energy intake and exercise duration during the 24 hours prior to meal consumption were not different by study period.

**Table 2:** Baseline data by study period.

Variables	Study Period 1 (n = 24)	Study Period 2 (n = 24)	P^1^
**TG (mg/dL)**^**2**^	66 (50.5–86.0)	66 (56.0–97.5)	0.37
**TG (mg/dL)**^**3**^	70.7 (58.5–85.5)	74.2 (62.7–87.8)	
**Log**_**e**_ **TG**^**4**^	4.3±0.4	4.3±0.4	0.40
**TC (mg/dL)**	157.5±29.5	160.0±32.4	0.29
**LDLC (mg/dL)**	87.5±24.9	90.5±27.3	0.16
**HDLC (mg/dL)**	52.6±11.8	53.7±11.9	0.18
**Non-HDLC (mg/dL)**	104.8±26.1	106.3±29.4	0.45
**TC:HDLC ratio**	3.1±0.7	3.1±0.7	0.63
**Body weight (kg)**	69.5±11.0	69.4±11.0	0.65
**EI over past 24-h (kcal)**	2366 (1628–3605)	2048 (1334–2839)	0.21
**ED over past 24-h (min)**	11.3 (0–50)	15 (0–43.8)	0.88

Abbreviations: TG, triglycerides; TC, total cholesterol; LDLC, low-density lipoprotein cholesterol; HDLC, high-density lipoprotein cholesterol; EI, energy intake; ED, exercise duration. ^1^P values indicate differences by period and were determined by Wilcoxon Signed Rank test for untransformed TG, EI, and ED and by paired t-test for the remaining variables. TG are shown as ^2^medians (25^th^ and 75^th^ percentiles), ^3^geometric means (95% CI), and ^4^log values. The data on EI and ED over past 24-h are shown as medians (25^th^ and 75^th^ percentiles) and the data on the remaining variables are shown as means and standard deviations.

### Lipids

**Fig 2:**
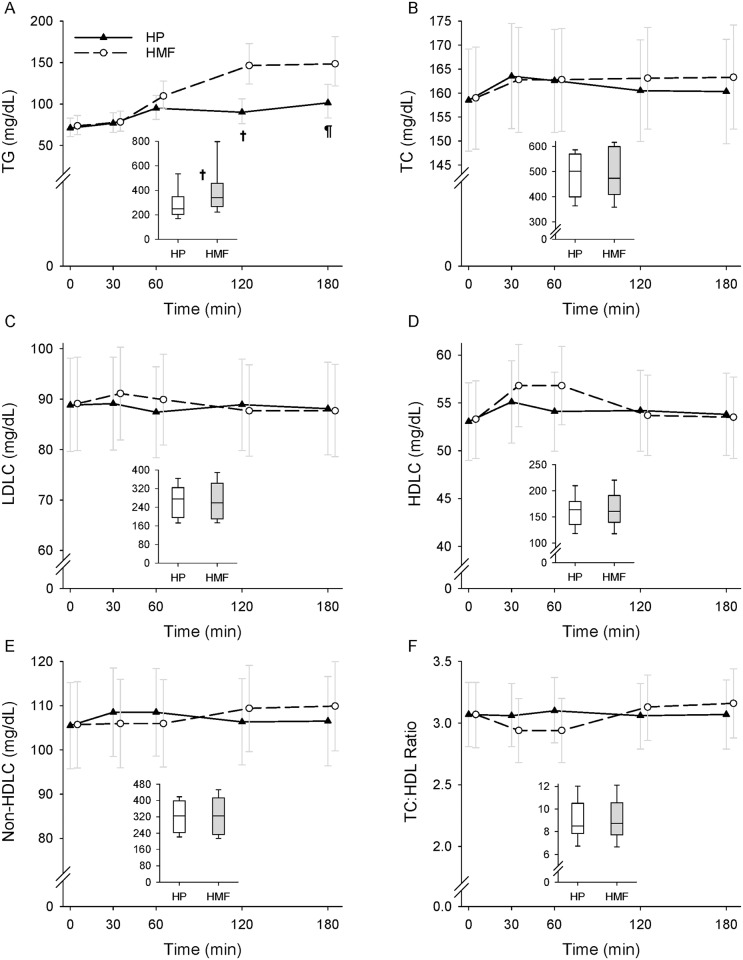
Postprandial lipid responses by meal condition. Triglyceride (TG) (A), total cholesterol (TC) (B), low-density lipoprotein cholesterol (LDLC) (C), high-density lipoprotein cholesterol (HDLC) (D), non-HDLC (E), and TC:HDLC (F) responses during a high-protein (HP) and a high-monounsaturated fat (HMF) meal condition in 24 participants. The line graphs show lipid responses over time as geometric means and 95% confidence intervals for TG and arithmetic means and 95% confidence intervals for the remaining variables. The box plots depict area under the curve (AUC) median (line within the box), 25^th^ and 75^th^ percentiles (lower and upper limits of the box), and 10^th^ and 90 percentiles (error bars). TG were log transformed before being analyzed. Mixed-effects model repeated measures analysis found a significant meal condition and time interaction effect for TG (p<0.0001), LDLC (p = 0.03), HDLC (p<0.0001), non-HDLC (p<0.0001), and TC:HDLC (p<0.0001) but not TC (p = 0.08). A significant time effect was found for TG (p<0.0001), TC (p = 0.001), LDLC (p<0.04), HDLC (p<0.0001), non-HDLC (p = 0.01), and TC:HDLC (p = 0.0002). There was no meal condition effect for any of the above variables except TG (p = 0.02). Repeated measures analysis showed significant difference in AUC by meal condition for TG (p<0.0001) but not for TC (p = 0.60), LDLC (p = 0.81), HDLC (p = 0.32), non-HDLC (p = 0.79), and TC:HDLC (p = 0.47). Differences between the HP and HMF meal conditions: ^¶^p<0.001; ^†^*p* < 0.0001.

The data on the postprandial lipid concentrations by meal composition and time are shown in Fig 2. The data on TG are shown as geometric means and 95% confidence intervals whereas the remaining data are shown as arithmetic means and 95% confidence intervals. AUC data on lipids are shown as box and whisker plots in Fig 2. The sequence in which the participants received HP or HMF had no effect on the lipid responses.

Mixed model repeated measures analysis showed a significant meal condition by time interaction (p<0.0001) effect on TG. TG concentrations (geometric means (95% CI)) were 71.1 (60.8–83.0), 76.9 (65.9–89.6), 95.0 (81.5–110.6), 90.1 (76.4–106.3), and 101.4 (83.1–123.8) mg/dL at baseline, 30 min, 60 min, 120 min, and 180 min, respectively, during the HP condition, and 73.8 (63.2–86.2), 78.5 (67.3–91.5), 109.7 (94.2–127.8), 146.5 (124.2–172.9), and 148.7 (121.9–181.4) mg/dL, respectively, during the HMF condition. The TG response was significantly lower during the HP compared to the HMF meal by 56.4 mg/dL (p<0.0001) at 120 min and 47.3 mg/dL at (p = 0.0006) 180 min. There was a significant time effect (p<0.0001). Compared to the corresponding baseline concentration, the TG concentration was significantly elevated by 5.8 (p = 0.002), 23.9 (p<0.0001), 19.0 (p<0.0001), and 30.3 (p<0.0001) mg/dL at 30, 60, 120, and 180 min, respectively, during the HP condition and by 4.7 (p = 0.01), 35.9 (p<0.0001), 72.7 (p<0.0001), and 74.9 (p<0.0001) mg/dL, respectively, during the HMF condition. TG AUC was also significantly lower during the HP compared to the HMF condition (geometric mean: 271.5 (95% CI: 228.7–322.4) vs. 365.7 (301.0–444.3) mg/dL*min; p<0.0001). The difference by meal condition in TG AUC was 94.2 mg/dL*min.

There was a significant treatment by time interaction effect on TG even after excluding the two participants on cholesterol lowering medications (p<0.0001). TG response in the participants not on cholesterol lowering medications was significantly lower on the HP compared to the HMF meal condition at 120 (geometric mean: 84.4 (95% CI: 72.1–98.7) vs. 138.2 (118.1–161.6) mg/dL; p<0.0001) and 180 (94.7 (77.8–115.1) vs. 138.2 (113.6–168.2) mg/dL; p = 0.0008) min. The difference by meal condition was 53.8 mg/dL at 120 min and 43.5 mg/dL at 180 min.

There was a significant time (p = 0.001) but no meal condition or meal condition by time interaction effect on TC. TC concentration was significantly elevated at 30 (mean difference: 3.8 (95% CI: 1.1–6.5) mg/dL; p = 0.007), 60 (3.8 (1.0–6.6) mg/dL; p = 0.009), 120 (4.1 (1.6–6.7) mg/dL; p = 0.002), and 180 (4.4 (1.7–7.1) mg/dL; p = 0.002) min following the HMF meal and at 30 (5.0 (2.3–7.8) mg/dL; p = 0.0004) and 60 (4.1 (1.3–6.9) mg/dL; p = 0.005) min following the HP meal compared to the respective baseline concentration. TC AUC was also not different by meal condition.

A significant meal condition by time interaction effect was found on LDLC (p = 0.03), HDLC (p<0.0001), non-HDLC (p<0.0001) and TC:HDLC ratio (p<0.0001) response. There was no meal condition effect for any of the above variables, however. The significant meal condition by time interaction effect was driven by the time effect (LDL: p = 0.04; HDLC: p<0.0001; non-HDLC: p = 0.01; TC:HDLC ratio: p = 0.0002). LDLC was significantly higher during the HMF meal condition at 30 min (mean difference: 2.0 (95% CI: 0.04–4.1) mg/dL; p = 0.046) compared to the baseline concentration but did not change in the HP meal condition. HDLC concentration was significantly elevated at 30 (mean difference: 3.5 (95% CI: 2.7–4.3) mg/dL; p<0.0001) and 60 (3.5 (2.4–4.6) mg/dL; p<0.0001) min during the HMF meal and at 30 (2.0 (1.2–2.9) mg/dL; p<0.0001) and 120 (1.1 (0.03–2.2) mg/dL; p = 0.04) min during the HP meal condition compared to the respective baseline concentration. Non-HDLC concentration was significantly higher at 120 (mean difference: 3.7 (95% CI: 2.0–5.5) mg/dL; p<0.0001)) and 180 (4.2 (2.2–6.2) mg/dL; p<0.0001) min during the HMF meal condition and at 30 (3.0 (0.8–5.2) mg/dL; p = 0.009) and 60 (3.0 (0.9–5.2) mg/dL; p = 0.006) min during the HP condition compared to the corresponding baseline concentration. TC:HDLC ratio was significantly lower at 30 (mean difference: 0.13 (95% CI: 0.08–0.18); p<0.0001) and 60 (0.13 (0.07–0.18); p<0.0001) min and higher at 120 (0.06 (0.03–0.09); p = 0.0006) and 180 (0.10 (0.06–0.14); p<0.0001) min compared to the fasting value during the HMF condition but did not change during the HP condition. There was no difference between the two meal conditions in AUC on LDLC, HDLC, non-HDLC, and TC:HDLC ratio.

### Lipoprotein particle numbers

**Fig 3:**
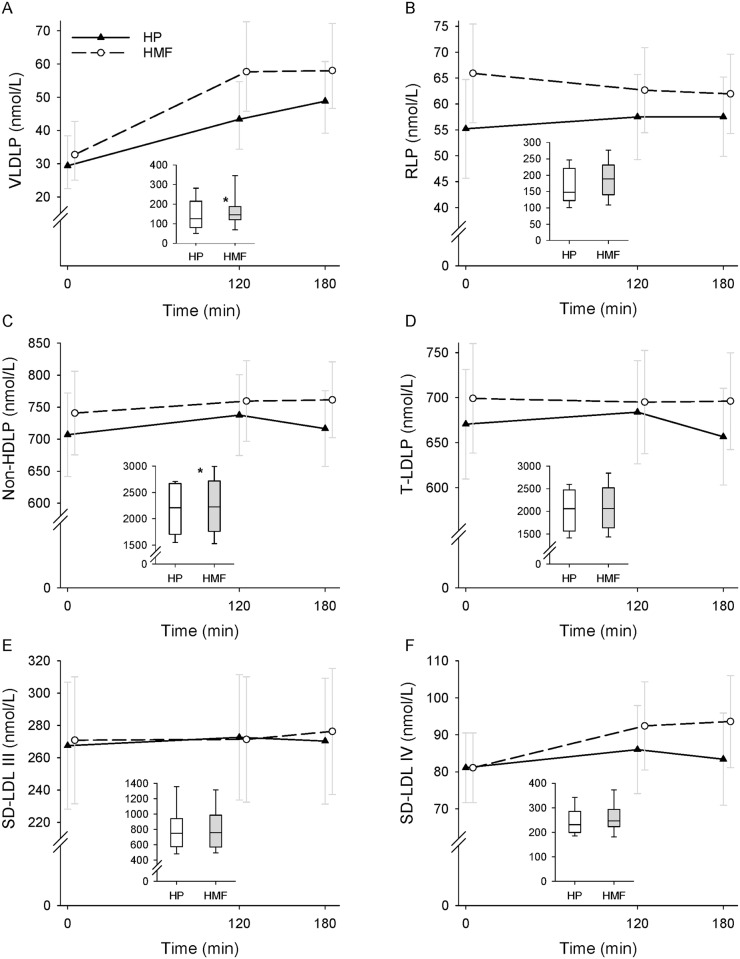
Postprandial lipoprotein particle responses by meal condition. Very low-density lipoprotein particle (VLDLP) (A), remnant lipoprotein particle (RLP) (B), non-high-density lipoprotein particle (non-HDLP) (C), total low-density lipoprotein particle (T-LDLP) (D), small-dense LDL III (SD-LDL III) particle (E), and small-dense LDL IV (SD-LDL IV) particle (F) responses during a high-protein (HP) and a high-monounsaturated fat (HMF) meal condition in 24 participants. The line graphs show geometric means and 95% confidence intervals for VLDLP and arithmetic means and 95% confidence intervals for the remaining variables. The box plots depict area under the curve (AUC) median (line within the box), 25^th^ and 75^th^ percentiles (lower and upper limits of the box), and 10^th^ and 90 percentiles (error bars). VLDLP were log transformed before being analyzed. Mixed-effects model repeated measures analysis found no meal condition and time interaction effect on VLDLP (p = 0.22), RLP (p = 0.17), non-HDLP (p = 0.27), T-LDLP (p = 0.13), SD-LDL III (p = 0.63), and SD-LDL IV (p = 0.12). There was no meal condition effect for any of the above variables (p = 0.22, p = 0.16, p = 0.31, p = 0.34, p = 0.90 and p = 0.36, respectively). Time effect was significant on VLDLP (p<0.0001), but not RLP (p = 0.94), non-HDLP (p = 0.14), T-LDLP (p = 0.52), SD-LDL III (p = 0.79) and SD-LDL IV (p = 0.07). AUC by meal condition was significantly different for VLDLP (p = 0.01) and non-HDLP (p = 0.04) but not RLP (p = 0.06), T-LDLP (p = 0.09), SD-LDL III (p = 0.93), and SD-LDL IV (p = 0.24). Differences between the HP and HMF meal conditions: *p < 0.05.

**Fig 4:**
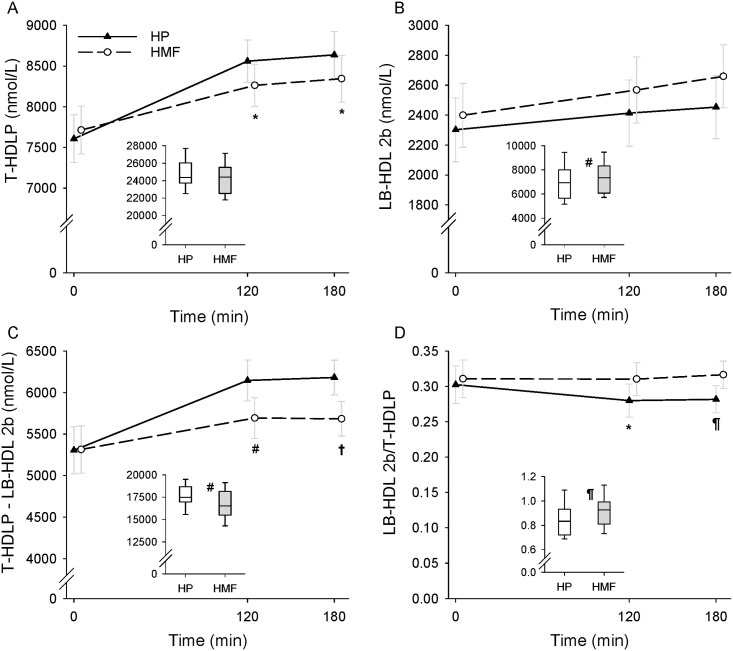
Postprandial lipoprotein particle responses by meal condition. Total high-density lipoprotein particle (THDLP) (A), large-buoyant HDL 2b (LB-HDL 2b) particle (B), T-HDLP—LB-HDL 2b (C), and LB-HDL 2b/T-HDLP ratio (D) responses during a high-protein (HP) and a high-monounsaturated fat (HMF) meal condition in 24 participants. The line graphs show arithmetic means and 95% confidence intervals. The box plots depict area under the curve (AUC) median (line within the box), 25^th^ and 75^th^ percentiles (lower and upper limits of the box), and 10^th^ and 90 percentiles (error bars). Mixed-effects model repeated measures analysis found a significant meal condition and time interaction effect for THDLP (p = 0.005), T-HDLP—LB-HDL 2b (p = 0.004), and LB-HDL 2b/T-HDLP (p = 0.01) but not LB-HDL 2b (p = 0.09). There was a significant meal condition effect for T-HDLP—LB-HDL 2b (p = 0.01), and LB-HDL 2b/T-HDLP (p = 0.04) and a significant time effect for THDLP (p<0.0001), LB-HDL 2b (p = 0004), and T-HDLP—LB-HDL 2b (p<0.0001), but not LB-HDL 2b/T-HDLP (p = 0.14). AUC by meal condition was significantly different for LB-HDL 2b (p = 0.006), T-HDLP—LB-HDL 2b (p = 0.002), and LB-HDL 2b/T-HDLP ratio (p = 0.0005) but not for T-HDLP (p = 0.07). Differences between the HP and HMF meal conditions: *p < 0.05; ^#^*p* < 0.01; ^¶^p<0.001; ^†^*p* < 0.0001.

The data on postprandial lipoprotein particle numbers by meal composition and time are shown in Fig 3 (VLDLP, RLP, non-HDLP, T-LDLP, SD-LDL III, and SD-LDL IV) and Fig 4 (T-HDLP, LB-HDL 2b, T-HDLP–LB-HDL 2b, and LB-HDL 2b/T-HDLP ratio). The data are presented as geometric means and 95% confidence intervals for VLDLP and arithmetic means and 95% confidence intervals for the remaining variables. The AUC data on VLDLP, RLP, non-HDLP, T-LDLP, SD-LDL III, and SD-LDL IV are shown in Fig 3 and those on T-HDLP, LB-HDL 2b, T-HDLP–LB-HDL 2b, and LB-HDL 2b/T-HDLP ratio are shown in Fig 4. The AUC data are shown as box and whisker plots.

There was no meal composition by time interaction effect on VLDLP, RLP, non-HDLP, T-LDLP, SD-LDL III, and SD-LDL IV response. Meal composition effect was also not significant on any of the above variables. Time effect was significant on VLDLP (p<0.0001), but not RLP, non-HDLP, T-LDLP, SD-LDL III and SD-LDL IV. VLDLP concentrations (geometric means (95% CI)) were 32.7 (25.1–42.7), 57.7 (45.7–72.7), and 58.0 (46.6–72.2) nmol/L at baseline, 120 min, and 180 min, respectively, during the HMF condition and 29.4 (22.5–38.4), 43.3 (34.4–54.7), and 48.8 (39.2–60.7) nmol/L, respectively, during the HP condition. VLDLP was significantly higher by 25.0 nmol/L (p<0.0001) at 120 min and 25.3 nmol/L (p<0.0001) at 180 min during the HMF condition and by 13.9 nmol/L (p<0.0001) at 120 min and 19.4 nmol/L (p<0.0001) at 180 min on the HP condition compared to the respective baseline value. AUC analysis showed that VLDLP was lower during the HP compared to the HMF meal condition (geometric mean: 150.6 (95% CI: 120.6–188.0) vs. 121.2 (91.7–160.2) nmol/L*min; p = 0.01) and the difference was 29.4 nmol/L*min. Non-HDLP AUC was also significantly lower on the HP compared to the HMF meal condition (mean difference: 89.4 (95% CI: 3.0–175.8) nmol/L*min; p = 0.04). There was no difference in RLP, T-LDLP, SD-LDL III, and SD-LDL IV AUC by meal condition.

There was a significant (p = 0.005) meal condition by time interaction effect on T-HDLP response. T-HDLP was significantly higher at 120 (mean difference: 297.3 (95% CI: 48.6–545.9) nmol/L; p = 0.02) and 180 (291.6 (15.8–567.5) nmol/L; p = 0.04) min in the HP versus the HMF condition. There was a significant time effect (p<0.0001). Compared to the corresponding baseline value, T-HDLP was significantly higher at 120 (mean difference: 548.9 (95% CI: 291.1–806.7) nmol/L; p<0.0001) and 180 (630.7 (351.8–909.5) nmol/L; p<0.0001) min in the HMF condition and at 120 (953.1 (695.3–1210.9) nmol/L; p<0.0001) and 180 (1029.2 (750.4–1308.0) nmol/L; p<0.0001) min in the HP condition. AUC on T-HDLP response was not different between the HP and the HMF conditions.

The meal condition by time interaction or meal condition effect for LB-HDL 2b did not reach statistical significance. There was a significant time effect (p = 0.0004). Compared to the corresponding fasting value, LB-HDL 2b was significantly higher at 120 (mean difference: 169.3 (95% CI: 76.5–262.0) nmol/L; p = 0.0006) and 180 (260.0 (152.0–368.0) nmol/L; p<0.0001) min in the HMF condition and at 120 (110.6 (17.9–203.3) nmol/L; p = 02) and 180 (151.5 (43.5–259.5) nmol/L; p = 0.007) min in the HP condition. AUC analysis revealed a significantly lower LB-HDL 2b response in the HP compared to HMF meal condition (mean difference: 432.4 (95% CI: 137.7–727.1) nmol/L*min; p = 0.006).

There was a significant (p = 0.004) effect of meal condition by time interaction effect on THDLP–LB-HDL 2b (a measure of small-dense HDL particles) response. T-HDLP—LB-HDL 2b response was significantly higher on the HP compared to the HMF condition at 120 (mean difference: 452.6 (95% CI: 177.4–727.9) nmol/L; p = 0.002) and 180 (496.8 (263.1–730.6) nmol/L; p<0.0001) min. There was also a significant time effect (p<0.0001). Compared to the corresponding baseline value, the response was significantly higher at 120 (mean difference: 379.7 (95% CI: 129.4–629.9) nmol/L; p = 0.004) and 180 (370.7 (104.9–636.4) nmol/L; p = 0.007) min during the HMF condition and at 120 (842.5 (592.3–1092.7) nmol/L; p<0.0001) and 180 (877.7 (611.9–1143.5) nmol/L; p<0.0001) min during the HP condition. T-HDLP—LB-HDL 2b AUC was significantly higher (mean difference: 917.2 (95% CI: 385.3–1449.0) nmol/L*min; p = 0.002) on the HP compared to HMF condition.

A significant (p = 0.01) meal condition and time interaction effect on LB-HDL2b/T-HDLP response was found. LB-HDL2b/T-HDLP was significantly lower on the HP compared to the HMF condition at 120 (mean difference: 0.03 (95% CI: 0.007–0.05); p = 0.01) and 180 (0.035 (0.015–0.055); p = 0.0009) min. LB-HDL2b/T-HDLP decreased significantly at 120 (mean difference: 0.02 (95% CI: 0.01–0.04) ratio; p = 0.005) and 180 (0.02 (0.004–0.04) ratio; p = 0.02) min compared to fasting value during the HP condition but did not change during the HMF condition. AUC analysis showed a significant meal condition effect on LB-HDL2b/T-HDLP. It was significantly (mean difference: 0.07 (95% CI: 0.035–0.11) ratio*min; p = 0.0005) lower during the HP versus HMF meal condition.

## Discussion

This is the first study to have examined the effect of a HP versus a HMF meal on postprandial lipid concentrations and lipid particle numbers. The mixed model repeated measures analysis or AUC analysis showed that the TG, non-HDLP, and VLDLP responses were lower on the HP compared to the HMF meal condition. The HP meal compared to the HMF meal condition, however, led to less favorable LB-HDL 2b (lower), T-HDLP—LB-HDL 2b (small-dense HDL particles) (higher), and LB-HDL2b/T-HDLP (lower) responses.

The TG AUC response in the present study was lower during the HP meal condition compared to the HMF meal condition. The TG response to the HMF meal in the present study is lower than that reported by Norata et al. [20, 21] and Minicocci et al. [20, 21]. This difference in response may be due to the fact that the two studies fed extremely high-fat meals containing 82% [20] and 69.1% [21] energy from fat, respectively, compared to 35.2% from fat in the present study. In addition, one of the studies [20] had participants with hypertriglyceridemia which may result in more enhanced postprandial TG response to a high-fat meal. In accordance with the present study, Appel et al. [2] reported lower fasting TG concentration after a HP compared to a HMF diet. According to a review paper by Khoury and Anderson [3], the lower TG response with high protein ingestion may be due to reduced lipid synthesis, slower stomach emptying, reduced synthesis and faster clearance of chylomicrons, and increased fat oxidation. A HP intake may suppress hepatic fat synthesis through increased levels of glucagon-like peptide-1 (GLP-1), a gut peptide [3, 22]. A recent study found that GLP-1 was higher following a HP meal compared to a HMF meal [23]. The HP meal in the present study had more yogurt than the HMF meal. Yogurt contains casein, whey, and branched chain amino acids (BCAA). Casein coagulates in the stomach and slows down gastric emptying [24]. Whey is associated with lower synthesis and faster clearance of chylomicrons possibly via increased lipoprotein lipase (LPL) activity [25]. BCAA supplementation reduces hepatic triglycerides due to upregulation of the transcription factor PPAR-α involved in fatty acid oxidation [26]. The postprandial TC, LDLC, HDLC, non-HDLC, and TC:HDLC ratio responses in the present study were not different between the two meal conditions. Luscombe-Marsh et al. [7] found no difference in fasting TC, LDLC, and HDLC concentration between a HP and HMF diet. Appel et al. [2] reported a lower fasting HDLC and TC on a HP compared to a HMF diet but no difference in fasting LDLC and non-HDLC between the two diets.

T-HDLP response was higher during the HP compared to the HMF condition at 120 and 180 min. The higher T-HDLP during the HP condition may be due to an increase in small-dense HDLP. In fact, T-HDLP–LB-HDL 2b, which would be made of mostly small-dense HDLP, was higher on the HP compared to the HMF condition. This was further confirmed by the results that LB-HDL 2b and LB-HDL 2b/T-HDLP were lower during the HP compared to HMF condition. Damsceno et al. [27] reported an increase in fasting large HDL concentrations following consumption of Mediterranean diets with either olive oil or nuts. Gill et al. [28], however, did not find a difference in HDL subtypes when saturated fat was replaced by monounsaturated fat content possibly because the percent energy from monounsaturated fat was only 13.7% in the HMF diet in this study compared to 20.7% in the present study. Wang et al. [29] reported a similar decrease in large HDLP across on an avocado-rich HMF diet and an oleic acid rich HMF diet compared to the baseline average American diet, possibly because the difference in monounsaturated fat content between the experimental diets and the baseline diet was less than the difference in the HMF and HP meal in the present study. A higher LB-HDL 2b on the HMF meal in the present study may be due to decreased production of cholesterol ester transfer protein (CETP). Jansen et al. [30] have shown that replacing a high-saturated fat diet with a high-monounsaturated fat diet decreases CETP concentrations. CETP transfers cholesterol esters from HDL to VLDL and triglycerides from VLDL to HDL. The triglycerides in the HDL are hydrolyzed by LPL leading to small dense HDL. A lower CETP concentration associated with a monounsaturated fat rich intake would reduce the conversion of large less-dense HDL to small-dense HDL.

Non-HDLP AUC and VLDLP AUC were lower in the HP compared to the HMF condition. There was no difference in RLP, T-LDLP, SD-LDL III, and SD-LDL IV between the HMF and HP conditions, however. Gill et al. [28] reported a lower SD-LDL but no change in VLDL subtypes when saturated fat was replaced by monounsaturated fat. Wang et al. [29] found a reduction in LDL particle number and small dense LDLC with an avocado rich HMF diet but not with an oleic acid rich HMF diet compared to the baseline average American diet. The same study [29] found no change in total VLDLP with either of the HMF diets compared to the baseline average American diet. Damasceno et al. [27] have reported a lower fasting TLDLP, SD-LDL, and large VLDL on a Mediterranean diet supplemented with nuts compared to baseline. The results from the above studies [27–29] are hard to interpret, however, because they were not designed to compare HMF to HP diets, and the values were fasting instead of postprandial values.

There are several limitations in the present study. Postprandial responses were measured in response to only one meal. It is not known if the results would be similar with subsequent meals of the same composition. The test meal was in a beverage form and it is not known if solid meals of the same composition would have a similar result. The test meals were also not adjusted for participant body surface area and this limits evaluation of inter-individual responses to the meals. The potential effect of not adjusting for body size in evaluating the intra-individual responses may be lessened by the cross-over design where each participant consumed both the meals containing the same energy content. Nevertheless, evaluation of both intra- and inter-individual responses to meals may be more appropriately addressed by taking body size into consideration when designing the test meals. There is a possibility that the postprandial duration of 3 hours in the present study may not have reflected peaks for certain outcomes measures. Adamska et al. examined the effect of a high fat meal on postprandial TG concentration over 4 hours and found that peak TG response occurred at 3 hours [31]. The participants in the study by Adamska et al. [31] were young and healthy, however, and the time to reach peak TG response to a high-fat meal in these subjects may not be applicable to older and less healthy individuals. Future studies should assess the lipid responses beyond 3 hours. Another potential issue with the 3-hour postprandial time period is that it may not be long enough to determine how the TG are distributed between chylomicrons, chylomicron remnants, VLDL, or VLDL remnants [32]. In addition, ApoB48 and ApoB100 were not determined. This may be a limitation because ApoB48 and ApoB100 indirectly reflect the TG rich lipoproteins, chylomicrons or chylomicron remnants and VLDL remnants. According to one study [33], most of the TG increase in the postprandial state is in VLDL remnants and not chylomicrons or chylomicron remnants, and VLDL remnants are linked to cardiovascular disease [33]. An additional limitation is that blood glucose and insulin were not assessed. Using the euglycemic, hyperinsulinemic, glucose clamp technique, Garg and colleagues [34] have shown that hyperinsulinemia or insulin resistance adversely affects VLDL-cholesterol, VLDL-triglycerides and TC:HDLC ratio. Since our participants had triglycerides in the normal range it is less likely that they had insulin resistance. In addition, the cross-over design of the study may have attenuated the impact of varying insulin concentrations on the intra-individual lipid responses to the meals. We a priori targeted completion of 24 study participants without a formal power estimation. However, the geometric mean differences for TG by meal condition at 120 (56 mg/dL; p<0.0001) and 180 (47 mg/dL; p = 0.0006) min were significant and would remain statistically significant even with conservative multiplicity adjustments. Although adequately powered for assessing TG differences, the sample size may have limited power for secondary study outcomes. Moreover, we analyzed many lipid variables without multiplicity adjustments for multiple endpoints.

The strengths of the study include a randomized cross-over design. The participants were diverse in age, gender, and race/ethnicity. The food intake and physical activity level on the day before the two study days were similar. Weight on the study days was also similar. The two meals had the same energy, carbohydrate, and sugar content. The protein, carbohydrate, and fat composition of both meals were within the guidelines set by the Institute of Medicine [15, 16] and therefore more suitable for consumption on a routine basis.

In conclusion, the postprandial TG, non-HDLP, and VLDLP responses were lower on the HP compared to the HMF meal condition. The HP meal led to less favorable postprandial LB-HDL 2b, T-HDLP—LB-HDL 2b (small dense HDLP) and LB-HDL2b/T-HDLP responses compared to the HMF meal. These results need to be confirmed by other studies and following multiple meals. The recommendation to consume a HP or HMF meal should be based on the lipid prolife of the participants.

## Supporting information

S1 File CONSORT checklist. CONSORT checklist submitted with manuscript.
(DOC)

S2 File Study protocol. Study protocol approved by the Institutional Review Board.
(DOCX)

S3 File Data. Study data in Excel format.
(XLSX)
