# Supplementary material for: Effect of meal composition on postprandial lipid concentrations and lipoprotein particle numbers: A randomized cross-over study
Source: PLoS One. 2017 Feb 21;12(2):e0172732. doi: 10.1371/journal.pone.0172732 (PMC5319704; doi:10.1371/journal.pone.0172732)
Supplement: S2 File — Study protocol approved by the Institutional Review Board. (DOCX) [file pone.0172732.s002.docx]

**
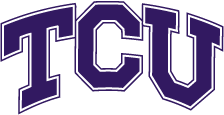
INSTITUTIONAL REVIEW BOARD**

**PROTOCOL REVIEW REQUEST**

The TCU Institutional Review Board (IRB) is responsible for protecting the welfare and rights of the individuals who are participants of any research conducted by faculty, staff, or students at TCU. Approval by the IRB must be obtained prior to initiation of a project, whether conducted on-campus or off-campus. While student research is encouraged at both the undergraduate and graduate level, only TCU faculty or staff may serve as Principal Investigator and submit a protocol for review.

Please submit this protocol electronically to [IRBFacultySubmit](mailto:IRBFacultySubmit@tcu.edu) (pdf preferred). Include the Protocol Approval Form as a word document with highlighted sections filled in. Also submit a consent document, HIPAA form if applicable, Protecting Human Research Participants Training certificates, recruitment materials, and any questionnaires or other documents to be utilized in data collection. A template for the consent document and HIPAA form, instructions on how to complete the consent, and a web link for the Protecting Human Research Participants Training are available on the TCU IRB webpage at [www.research.tcu.edu](http://www.research.tcu.edu). Submission deadline for protocols is the 15^th^ of the month prior to the IRB Committee meeting.

1. **Date:** 8/14/15
2. **Study Title:** The effect of meal composition on blood lipids
3. **Principal Investigator (must be a TCU faculty or staff):** Meena Shah, Ph.D.
4. **Department:** Kinesiology
5. **Other Investigators: List all faculty, staff, and students conducting the study including those not affiliated with TCU.**

Manall Jaffery, Joel Mitchell, Ph.D., Jonathan Oliver, Ph.D., Melody Phillips, Ph.D., Andreas Kreutzer, Lauren Nelson, Kylie von Richter, Angela Baleztena, Audrianna Ebel, Chris Martinez, Michael Levitt, Cheryl Haston, Adam Gloeckner.

1. **Project Period:** 09-15-15 to 9-14-16
2. **If you *have* external funding for this project –**

**Funding Agency:** TCU RCAF grant **Project #:** N/A **Date for Funding:** 6/1/15

1. **If you *intend to seek/are seeking* external funding for this project –**

**Funding Agency:** N/A **Amount Requested From Funding Agency:** N/A

**Due Date for Funding Proposal:** N/A

1. **Purpose: Describe the objectives and hypotheses of the study and what you expect to learn or demonstrate:**

The purpose of this study is to compare the effect of a high-monounsaturated fat meal versus a high-protein meal on postprandial (after a meal) blood concentrations of total cholesterol (TC), high-density lipoprotein cholesterol (HDLC or good cholesterol), low density lipoprotein cholesterol (LDLC or bad cholesterol), triglycerides (TG - the predominant fat in the body), non-HDL cholesterol (TC - HDLC), TC/HDLC ratio, and LDLC/HLDC ratio.

It is hypothesized that a high-protein meal will generally result in healthier postprandial blood lipid concentrations than a high-monounsaturated fat meal of the same energy content.

1. **Background: Describe the theory or data supporting the objectives of the study and include a bibliography of key references as applicable.**

Heart disease is the leading cause of death in the US^1^. Major risk factors for heart disease include high blood concentration of TC, LDLC, TG, and non-HDLC, low concentration of HDLC, and high TC/HDLC ratio and LDL/HDLC ratio^2^.

Blood lipids are influenced by diet composition. Studies show that replacing carbohydrates with monounsaturated fat lowers TG and increases HDLC^3^, replacing carbohydrates with protein lowers TG^4^, and replacing saturated fat with monounsaturated or polyunsaturated fat lowers LDLC^5^. What is not well understood, however, is how a high-protein intake affects blood lipids compared to a high-monounsaturated fat intake.

Only two studies have compared the effect of a diet rich in monounsaturated fat with a diet rich in protein on blood lipids. Appel et al.^6^ reported that a high-protein diet significantly lowered fasting blood TC, TG, and HDLC and tended to lower non-HDLC compared to a high-monounsaturated fat diet of similar calorie content. There was no difference in LDLC between the two diets. Luscombe-Marsh et al.^7^, on the other hand, found no difference in fasting TC, LDLC, HDLC, and TG following a hypo-caloric (low calorie) high-protein versus a hypo-caloric high-monounsaturated fat diet. The two studies had several limitations. In the study by Appel et al.^6^, the high-protein diet was only modestly high in protein (25% energy from protein) and the study was conducted in only hypertensive or pre-hypertensive subjects and the results may not be generalizable. In the study by Luscombe-Marsh et al.^7^, the carbohydrate content of the two diets was low (35-36% energy from carbohydrate) and unsustainable over the long-run and polyunsaturated fat intake was higher in the high-monounsaturated fat diet compared to the high-protein diet. Moreover, the subjects in both diet groups lost weight^7^. This makes it difficult to distinguish the effect of diet composition from weight loss on lipids. In addition, both the above studies^6,7^, measured just the fasting lipid responses to the two diets and not the postprandial responses. Postprandial lipid responses are important to examine since they are associated with heart disease^8,9^ and most individuals in Western countries are in a postprandial state for most of the day^10,11^.

**References**

1. Kochanek KD et al. Mortality in the United States, 2013. NCHS Data Brief Dec 2014;178:1-8.
2. National Cholesterol Education Program. Executive summary of the third report of the National Cholesterol Education Program (NCEP) expert panel detection, evaluation, and treatment of high blood cholesterol in adults (Adult Treatment Panel III). J Am Med Assoc 2001;285:2486-2497.
3. Shah M et al. Effect of a high-carbohydrate diet versus a high-cis-monounsaturated fat diet on lipid and lipoproteins in individuals with and without type 2 diabetes mellitus. Nutrition Research 2004;24:969-979.
4. Wycherley TP et al. Effects of energy-restricted high-protein, low-fat compared with standard-protein, low-fat diets: a meta-analysis of randomized controlled trials. AJCN 2012;96:1281-98.
5. Grundy SM, Denke MA. Dietary influences on serum lipids and lipoproteins. J Lipid Res 1990;37(7):119-72.
6. Appel LJ et al. Effects of protein, monounsaturated fat, and carbohydrate intake on blood pressure and serum lipids. JAMA 2005;294:2455-64.
7. Luscombe-Marsh ND. Carbohydrate-restricted diets high in either monounsaturated fat or protein are equally effective at promoting fat loss and improving blood lipids. AJCN 2005;81:762-72.
8. Simons LA et al. Chylomicrons and chylomicron remnants in coronary artery disease: a case-control study. Atherosclerosis 1987;65:181-189.
9. Bansal S et al. Fasting compared with nonfasting triglycerides and risk of cardiovascular events in women. J Am Med Assoc 2007;298:309-316.
10. Anderson JW et al. Postprandial serum glucose, insulin, and lipoprotein responses to high- and low-fiber diets. Metabolism 1995;44:848-854.
11. Garg A et al. Effects of varying carbohydrate content of diet in patients with non-insulin-dependent diabetes mellitus. J Am Med Assoc 1994;271:1421-1428.
12. **Subject Population: Describe the characteristics of the participant population including the inclusion and exclusion criteria and the number of participants you plan to recruit:**

Up to sixty subjects between the ages of 19-65 y will be recruited for the study. Exclusion criteria will include use of medications or supplements that affect lipid levels, body weight, or blood glucose, following a weight loss diet, being a vegan, smoking, heavy alcohol use (>14 and >7 drinks/week for men and women, respectively), pregnancy, lactation, severe depression, eating disorders, presence of liver, kidney, gastrointestinal, adrenal, or untreated thyroid disease, diabetes, lactose intolerance, documented malabsorption, or bowel surgery that affects absorption.

1. **Recruitment Procedure: Describe your recruitment strategies including how the potential participants will be approached and precautions that will be taken to minimize the possibility of undue influence or coercion. Include copies of the recruitment letters, leaflets, etc. in your submission.**

The subjects will be recruited by placing fliers around the TCU campus and the surrounding communities such as medical clinics and churches, by advertisements placed in TCU newsletters, local newspapers, and the social media, announcements made in the classrooms, by e-mail, and by word of mouth. Permission from the medical clinics, churches, or any community entity will be sought before leaving any fliers in those establishments. A recruitment flyer has been included in the Appendix.

To prevent the possibility of undue influence or coercion, potential participants will be informed that participation is completely voluntary and that they may withdraw at any time without penalty.

1. **Consenting Procedure: Describe the consenting procedure, whether participation is completely voluntary, whether the participants can withdraw at any time without penalty, the procedures for withdrawing, and whether an incentive (describe it) will be offered for participation. If students are used as participants, indicate an alternative in lieu of participation if course credit is provided for participation. If a vulnerable population is recruited, describe the measures that will be taken to obtain surrogate consent (e.g., cognitively impaired participants) or assent from minors and permission from parents of minors.**

Participants will read and sign a TCU IRB approved consent document prior to participation. Participation is completely voluntary and participants may withdraw at any time without penalty. A participant can withdraw by informing either Manall Jaffery or Meena Shah that he/she does not wish to continue participation. Manall Jaffery can be reached by telephone (469 426-4030), e-mail (m.jaffery@tcu.edu), or in person. Meena Shah can be reached by telephone (817 257-6871), e-mail (m.shah@tcu.edu), or in person. There will be no incentive to participate in this study. Students will not receive class credit for participating in the study. Surrogate consent or assent from minors will not be necessary because vulnerable populations and minors will not be recruited.

1. **Study Procedures: Provide a chronological description of the procedures, tests, and interventions that will be implemented during the course of the study. Indicate the number of visits, length of each visit, and the time it would take to undergo the various tests, procedures, and interventions. If blood or tissue is to be collected, indicate exactly how much in simple terms. Flow diagrams may be used to clarify complex projects.**

Each participant will be instructed to report to the Metabolic/Exercise Physiology Laboratories on three separate days. The first day will be used for screening and the second and third days will serve as study days. On all three days, the participants will report to the labs after a 12-hour overnight fast. Subjects will also be asked to abstain from any exercise for 24 hours prior to each study day.

Measures taken during the first screening day will include completion of pregnancy questionnaire (see Appendix) and optional urine pregnancy test in women of child bearing age. Women who report that they are pregnant or think that there is a possibility of pregnancy or test positive on the optional urine pregnancy test will be not be allowed to undergo DEXA and will be excluded from the study. Other measures during screening will include a fasting blood draw, measurement of height, weight, waist/hip circumferences, and body composition, and collection of demographic, behavioral, and health information. A fasting blood sample of 20 ml (4 teaspoons) will be drawn and used to determine complete cell blood count, blood lipids, hormones that affect blood glucose, blood glucose, and markers of inflammation. Data on demographics, behavioral, and health information will also be collected via questionnaires (see Appendix). Percent body fat and fat distribution will be assessed by dual energy x-ray absorptiometry (DEXA). Eligible subjects will be asked to remove all metal objects including clothing containing metal and wear light clothing with no metal objects. The subject will then be instructed to lie on the DEXA table (the table is padded) for about 10-20 min while the scanner passes over the subject’s body. The subject will be exposed to two low dose x-rays during this procedure. The procedure will generate an image which will be analyzed to estimate the amount of total body fat, lean tissue, and bone mass. The radiation exposure from this procedure is very low and similar to the amount received from the sun while flying from coast to coast. The first screening visit will take approximately 50 minutes to complete.

The information collected during the screening visit will be used to determine if the subject is eligible for the study. Some of the questionnaires on health may indicate presence of eating disorders. Subjects with eating disorders will not be included in the study but will be referred to the TCU Health Clinic or the eating disorders hotline (1-800-411-7081) at Texas Health Resources. Subjects with any health condition will be asked to talk to their personal physician.

The two subsequent visits or study days will be scheduled between 6 and 10 a.m. and at least 4 days apart. Each participant will be asked to keep his/her food and drink intake the same on the day before the study days and come to the lab in a 12 hour fasted state. The subject will also be asked to abstain from any exercise for 24 hours prior to the two study days. A study investigator will ensure that the above study requirements are met by asking the participant to recall her/his food intake and physical activity levels over the past 24 hours. After ensuring that the participant has complied with the above requirements, a venous catheter will be inserted into the antecubital vein of the participant’s preferred arm and 20 ml (4 teaspoons) of fasting blood sample will be taken while the subject is lying down. The participant will then be randomly assigned to consume either a high-monounsaturated fat meal or a high-protein meal. The high-monounsaturated fat meal will contain 35-40% energy from fat (mainly monounsaturated fat), 10-15% energy from protein, and 45-60% energy from carbohydrate. The high-protein meal will contain 30-35% energy from protein, 15-20% from fat, and 45-60% from carbohydrate. The subjects will be instructed to consume all the food at each meal within 20 minutes while seated. A postprandial blood sample (20 ml) will be taken at 30, 60, 120, and 180 minutes after the meal begins. Saline flushes will be provided after each sample to keep the catheter clean and viable. The subjects will be instructed to drink water throughout the study to prevent dehydration. The participants will be also be asked to assess their feeling of hunger, fullness, desire to eat, and thirst immediately before the meal begins and at 30, 60, 120, and 180 minutes after the meal begins using the attached questionnaire. Meal palatability will be assessed, using the attached questionnaire, at 1 minute after the meal begins and upon the termination of the meal. Blood pressure will be assessed immediately before the meal begins and at 30, 60, 120, and 180 minutes after the meal begins. Only the arm without the catheter will be used for this measure. During the subsequent study day, the subjects will undergo all the above study day procedures but will be fed the meal with the alternate composition. Both meals will contain the same calorie content for each subject. The amount of time taken during each study day will be about 200 minutes.

The blood samples will be processed and stored at minus 80**°**C until analysis. Blood samples will only be drawn by qualified investigators including Drs. Mitchell, Phillips, Shah, and Oliver, Manall Jaffery, and Andreas Kreutzer. Other investigators on the investigator list will be allowed to draw blood only after they receive training and approval from Drs. Mitchell and Phillips. Blood concentrations of lipids, inflammatory markers, and hormones that control blood glucose will be assessed using ELISA method following manufacturer instructions for each measure. Blood glucose concentrations will be assessed enzymatically, in triplicate, using a spectrophotometer.

The total amount of time required for the 3 visits will be about 450 minutes (7.5 hours).

1. **Data Analyses: Describe how you will analyze your data to answer the study question.**

A two-factor (2 x 5, meal composition by time) repeated measures analysis will be used to assess the effect of the meal composition (high-monounsaturated fat meal and high-protein meal) and time (before meal ingestion, and at 30, 60, 120, and 180 minutes from when meal begins) and the interaction between these factors on blood concentrations of lipids, inflammatory markers, hormones that control blood glucose, blood glucose, and ratings of hunger, fullness, desire to eat, and thirst, and blood pressure. Differences in these outcome variables by meal composition and time will be analyzed by least square means. The area under the curve for these variables will also be computed using the trapezoidal rule and analyzed with repeated measures analysis models. The above analyses will also be performed with age, sex, and percent body fat adjustments. All data will be analyzed using SPSS version 18.0 (SPSS Inc., Chicago, IL).

1. **Potential Risks and Precautions to Reduce Risk: Indicate any physical, psychological, social, or privacy risk which the subject may incur. Risk(s) must be specified. Also describe what measures have been or will be taken to prevent and minimize each of the risks identified. If any deception is to be used, describe it in detail and the plans for debriefing.**

Risks of the blood sampling procedure include bruising, hematoma, dizziness, fainting, pain upon needle stick, and the remote risk of infection. Redness or swelling at the site of needle stick or fever may indicate a sign of an infection. The subjects will be asked to see their personal physician or health center if an infection develops at the site of the blood draw. The above risks will be minimized by having a trained investigator clean the site of catheter insertion using alcohol swabs and obtain blood samples using sterile, single-use, disposable supplies while the participant is supine.

The primary risk associated with dual-energy x-ray absorptiometry (DEXA) is radiation exposure. The radiation dose for one DEXA scan to measure body composition and bone density is 1.5 millirem.  This is similar to the amount of natural background radiation a person would receive in one month while living in north TX or less than what a person would be exposed to during an airplane flight from New York City to Los Angeles (2 to 5 millirem). The Radiological Society of North America, Inc. considers this dosage to represent a negligible level of additional risk of disease caused by radiation. The maximal permissible x-ray dose for non-occupational exposure is 500 millirem per year. However, radiation may be harmful to the developing fetus and DEXA will not be performed on pregnant women. Immediately upon arrival for screening, we will ask women of child-bearing age to complete a questionnaire on pregnancy (see Appendix). Women who report that they are pregnant or think there is a possibility that they are pregnant will be excluded from the study. All women will be given the option of a urine pregnancy test. Women who test positive based on the urine test will be excluded from the study. A woman who tests positive on a pregnancy test will be informed in private setting. Risks will be further reduced by having only certified technicians operate the DEXA machine. **The above precautions include all the previously approved guidelines by the IRB on how to minimize the risk associated with DEXA.**

The participant may feel uncomfortable while height, weight, and waist and hip circumference are being measured. This will be minimized by taking these measurements in the Metabolic Lab with only one or two investigators present in the room. The participant may feel self-conscious while completing the questionnaires. This will be minimized by having the participant complete most of the questionnaires in a private setting.

The participants may feel self-conscious when consuming the meals. To make the participants feel more at ease, the investigators will allow the subjects to listen to music of their choice and not overtly watch them while they eat.

Additionally, there is the potential for food borne illnesses which will be minimized by following safe food handling procedures.

1. **Procedures to Maintain Confidentiality: Describe how the data will be collected, de-identified, stored, used, and disposed to protect confidentiality. If protected health information is to be re-identified at a later date, describe the procedure for doing so. All signed consents and hard data must be stored for a minimum of 3 years in a locked filing cabinet (and locked room) in the principal investigator’s office, lab, or storage closet at TCU. Your professional society may recommend keeping the materials for a longer period of time.**

All data will be stored in a locked cabinet in the Metabolic Lab. The data will be stored for at least 3 years. Electronic data will be stored without identifiers in a password protected computer. The only people with access to the data (both electronic and hard copies) will be the investigators. The data will be presented and published without any identifying information.

1. **Potential Benefits: Describe the potential benefits of the research to the participants, to others with similar problems, and to society.**

No study to date has compared the effect of a high-monounsaturated fat versus a high-protein meal on postprandial concentrations of blood lipids. Examining this is important since most individuals in Western countries are in a postprandial state for most of the day and postprandial lipid levels are associated with cardiovascular disease. In addition, many Americans consume high-protein meals indicating the need to investigate the effect of high-protein intake on postprandial lipid levels. Moreover, comparing a high-protein intake to a high-monounsaturated fat intake is appropriate since the latter is recommended to lower the risk for cardiovascular disease.

The participant may benefit from this study by learning which meal optimizes their lipid profile. The participant may also benefit from the information on body composition and bone density obtained from DEXA.

1. **Training for Protecting Human Research Participants: Submit training certificates for all the study investigators. The training link is available on the TCU IRB webpage at** [**www.research.tcu.edu**](http://www.research.tcu.edu)**.**
2. **Check List for the Items That Need to be Submitted: Please combine all the files into one pdf document before submitting the materials electronically to the IRB. To prevent any delay in the approval of your protocol, use the most recent template for the protocol, consent document, and HIPAA form by downloading them from** [**www.research.tcu.edu**](http://www.research.tcu.edu) **each time you prepare your materials.**

| 1. Protocol | X |
| --- | --- |
| 1. Consent document | X |
| 1. HIPAA form if applicable | X |
| 1. Protecting Human Research Participants Training certificate for each investigator | X |
| 1. Recruitment fliers, letters, ads, etc. | X |
| 1. Questionnaires or other documents utilized in screening and data collection | X |
